# A Novel Pathogen Capturing Device for Removal and Detection

**DOI:** 10.1038/s41598-017-05854-4

**Published:** 2017-07-17

**Authors:** Gwangseong Kim, Horatiu Vinerean, Angelo Gaitas

**Affiliations:** 1 Kytaro, Inc., Miami, FL 33199 USA; 2 Department of Electrical and Computer Engineering, Florida International University, Miami, 33199 Florida USA; 3 Office of Research and Economic Development, The Office of Laboratory Animal Research, Florida International University, Miami, 33199 Florida USA

## Abstract

A simple technique that employs an antibody coated polydimethylsiloxane tube is used for effective capturing of bloodborne and foodborne pathogens. By recirculating the entire sample through the antibody coated tube, accumulation of target pathogens is achieved, thereby delivering a higher concentration of pathogens in a small volume. The described method can provide an effective and economical solution to microbiology techniques that rely on enrichment, thereby expediting diagnostics. Using this method 80.3 ± 5.6% of *Staphylococcus aureus* with a starting concentration of ~10^7^ CFU/mL and 95.4 ± 1.0% of Methicillin-resistant *Staphylococcus aureus* with starting concentration of ~10^4^ CFU/mL were removed from 5 mL blood in a few hours. This concept was extended to live rats with an induced bloodstream *S*. *aureus* infection. A reduction of two orders of magnitude in the bacterial load of the rats was observed within a few hours. The same technique was used to capture a food pathogen, *Salmonella typhimurium*, with starting concentrations as low as ~10^0^ CFU, from 100 or 250 mL of culture broth within similar timeframes as above. The feasibility for food pathogen testing applications was additionally confirmed by capturing and detecting *S*. *typhimurium* in ground chicken and ground beef.

## Introduction

In recent years, there have been considerable efforts to develop devices and methods for capturing of pathogens in fluids such as blood and other liquid media (for example food matrices and water)^1–4^. These efforts are motivated by the need to quickly capture pathogens for detection of bloodborne infections^5–7^, for detection of pathogens in food products^8–11^, or even for therapeutic purposes^12–18^. Some indicative examples include extracorporeal blood circulation methods to capture target pathogens (e.g. circulating tumor cells) using immunocapturing techniques reported for *in vivo* diagnostics and therapies^5–7^ and immunomagnetic concentration technologies for food pathogens^11^. Recently, a microfluidic device that relies on immunomagnetic separation (IMS) technology using an engineered antibody^13^ was used to remove bacteria and toxins from blood. A hemofiltration cartridge was developed using the same engineered antibody^12^. It is evident that there is a multitude of applications and the specific parameters for each may vary (for instance some applications require high volume pathogen removal, such as food pathogen testing and environmental testing^1, 2^, while others require ability to capture low quantities of pathogens, such as blood infection diagnosis and circulating tumor cell detection^4, 6, 7^). However, the overarching need for a simple and inexpensive way to remove pathogens from liquid media remains due to the common needs these applications share, which are: ability to capture the majority of the pathogens present in the liquid media irrespective of the total quantity of the pathogens and the media volume, ability to process samples with complex constituents, ability to perform capturing in a rapid manner. Unfortunately, current technologies fail to simultaneously address all these concerns.

**Figure 1:**
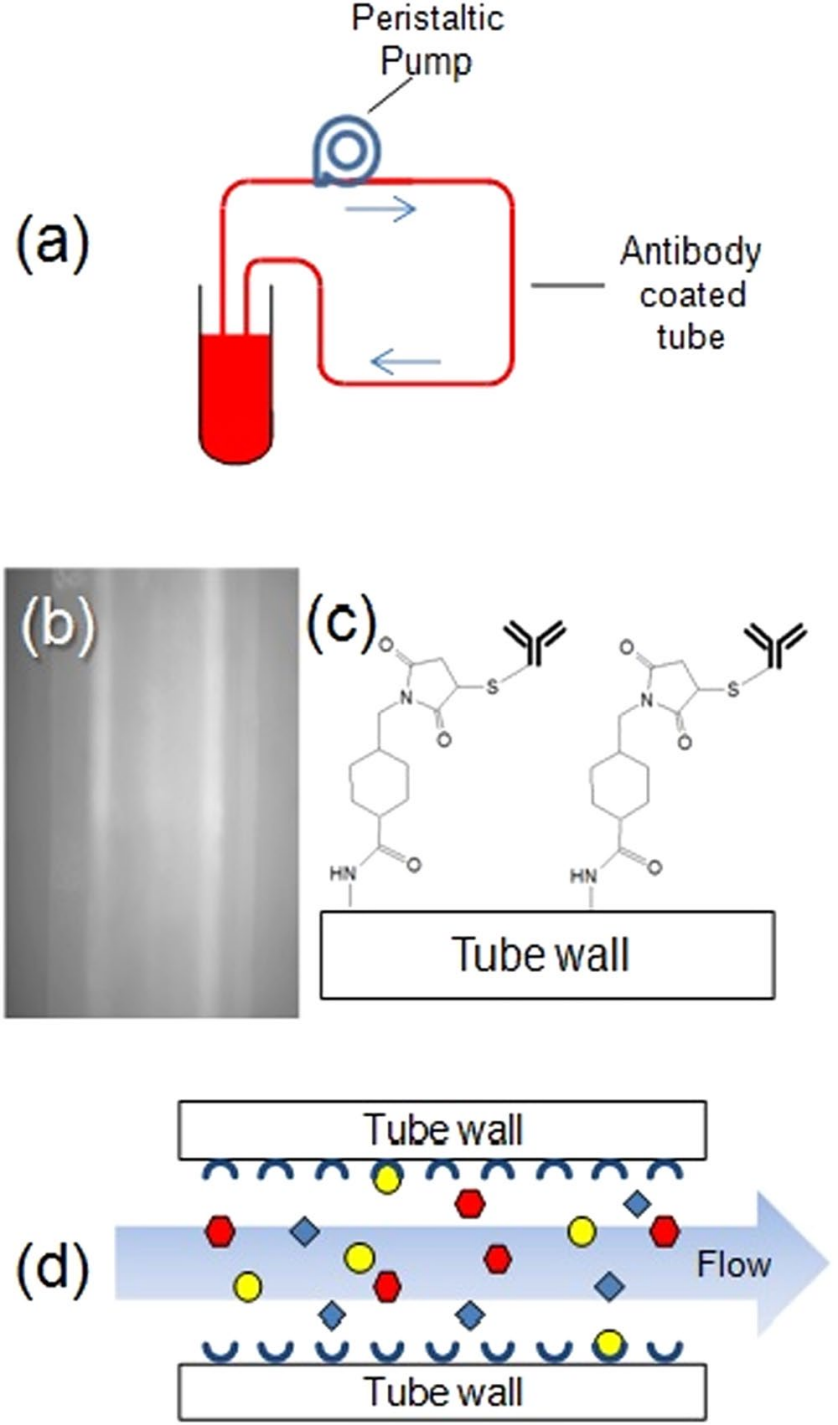
Principle of tube capturing by constant flow (**a**) Diagram of experimental setup. (**b**) Fluorescence image *S*. *aureus* antibody coated tube, confirmed by staining with Alexa 488 labeled secondary antibody. (**c**) Diagram of antibody coated tube surface showing the tube chemistry. (**d**) Selective pathogen capturing inside tube.

In this manuscript, a simple method that addresses the aforementioned challenges is presented. The key feature of this method is the recirculation of the liquid media through an antibody conjugated polymer tube (Fig. 1b,c) using a simple arrangement that includes a peristaltic pump (Fig. 1a). During the flow through the tube the pathogens are captured by antibodies or other adhesion molecules. This capture and subsequent continuous flow of the sample promotes the accumulation of the target organism inside the tube (Fig. 1d). Furthermore, several tubes with antibodies can be used enabling capture of multiple kinds of pathogens simultaneously. In this manuscript, capturing of microbial pathogens is demonstrated such as *S*. *aureus* (gram positive), MRSA (gram positive) from blood at high concentration *in-vitro* and *in-vivo* and *S*. *typhimurium* (gram negative) from culture media and food matrices in very low concentrations (about 10^0^ CFU in 250 mL in pure culture and about 25 CFU in 250 mL food matrix). Positive detection with immunofluorescence and PCR proves that the pathogens were captured. The principle was confirmed in food matrices (ground chicken and ground beef). Various applications of this technology for pathogen reduction, infection diagnosis and food pathogen testing are discussed.

## Results and Discussion

### Bacterial capturing in blood

**Figure 2:**
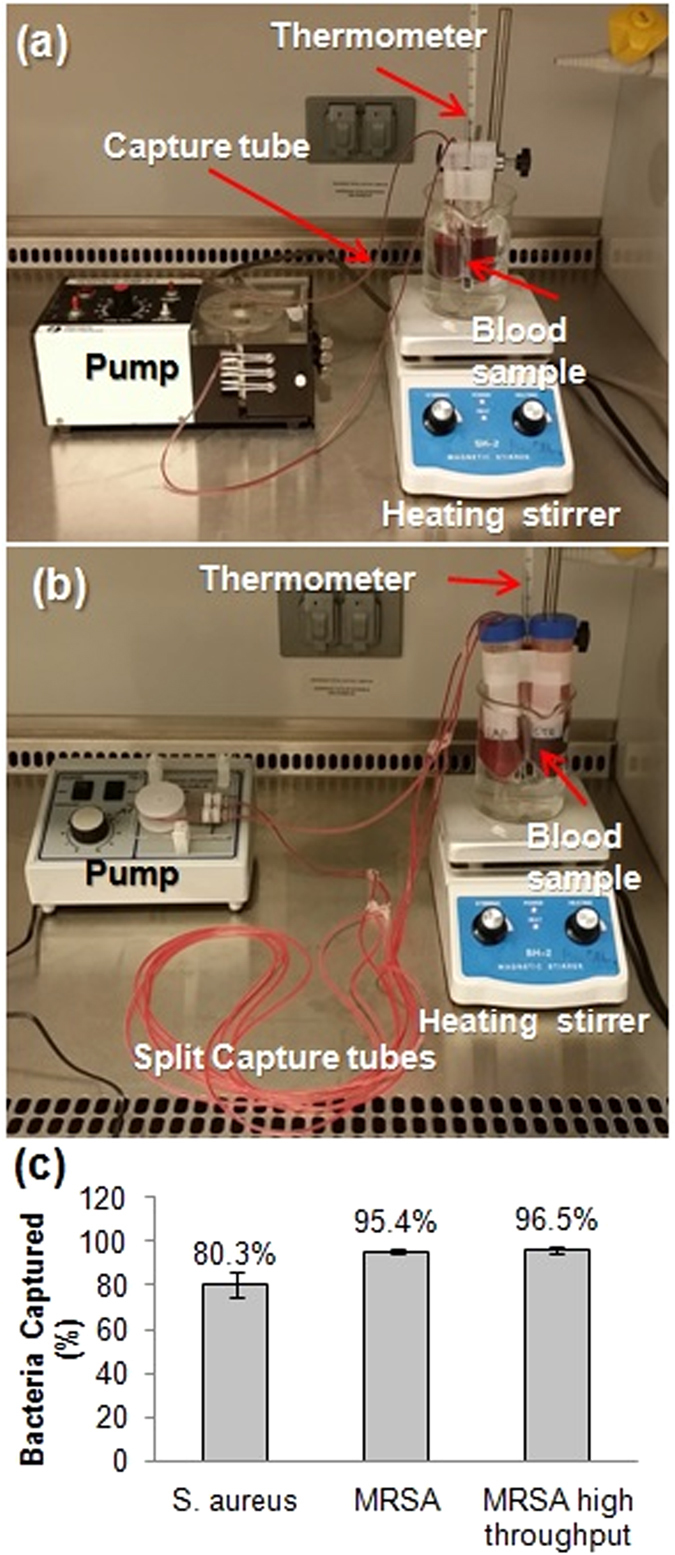
*In vitro* tube capturing. (**a**) Experimental set-up of 5 mL blood volume at 0.5 mL/min flow rate. (**b**) Experimental set-up for the high throughput experiments of 20 mL blood volume at 5 mL/min flow rate. (**c**) Summary of capturing efficiency presented in % captured bacteria compared to controls. 80.3 ± 5.6% of S. aureus with a starting concentration of ~10^7^ CFU/mL was removed from 5 mL blood in 5 hours (n = 5), 95.4 ± 1.0% of MRSA from a starting concentration of ~104 CFU/mL was removed from 5 mL blood in 5 hours for (n = 5). 96.5 ± 1.6% of MRSA from a starting concentration of ~10^4^ CFU/mL was removed from a high throughput condition, 20 mL blood at 5 mL/min flow rate in 5 hours (n = 4).

The study was initiated with *S*. *aureus* in high starting concentration (10^7^ CFU/mL) to test whether this technique can achieve effective capturing of bacteria in *in vitro* conditions. After confirmation of ability to capture pathogens, an antibiotic resistant strain of *S*. *aureus*, MRSA, was used in clinically relevant concentration ranges (~10^4^ CFU/mL)^19, 20^. The experimental setup is shown in Fig. 2a. Tube capturing for *S*. *aureus* yielded an average of 80.3 ± 5.6% reduction compared to the control values (n = 5) as shown in Fig. 2c. Tube capturing for MRSA resulted in an average 95.4 ± 1.0% reduction (n = 5), as summarized in Fig. 2c (a full data set for this study can be found in the Supplementary information, Fig. S1, Tables S1 and S2). The capture and detection by real time PCR of MRSA in clinically relevant low concentrations was confirmed (Supplementary information S1.1. S1.2 and S2). Clogging was not observed in these experiments.

### *In vivo* capturing *S*. *aureus* in infected rats

**Figure 3:**
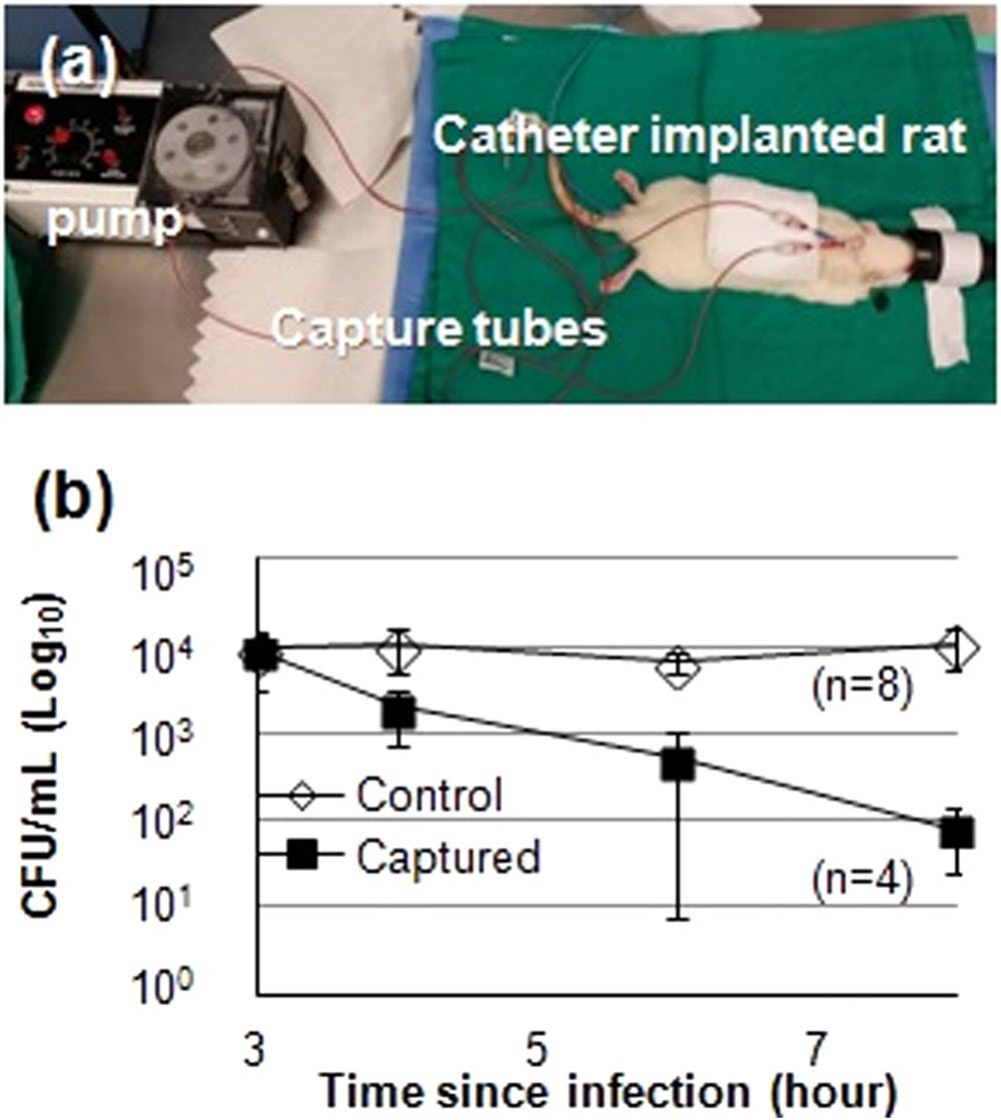
*In vivo* tube capturing results. (**a**) Experimental set-up. (**b**) Average bacterial density in collected blood from rats over time (y axis in logarithmic scale).

Extracorporeal blood circulation methods have been used to capture target pathogens (e.g. circulating tumor cells or bacteria) for *in vivo* diagnostics and therapeutic purposes^5–7, 12, 13^. The simple tube arrangement described in this manuscript was tested for *in vivo* pathogen removal in a rat model. Wistar rats were infected by intraperitoneal (IP) injection with 2 × 10^10^ CFU of *S*. *aureus*. After 3 hours from the injection, extracorporeal circulation through a 240-cm antibody coated tube was performed for 5 hours at 0.5 mL/min flow rate (Fig. 3a). At 5 hours, a reduction of over two orders of magnitude was observed in bacterial concentration. Bacteria in the bloodstream were reduced from about 0.9 × 10^4^ CFU/mL to 0.8 × 10^2^ CFU/mL on average (n = 4). Rats in the control group showed nearly constant presence of *S*. *aureus* (ranging from about 0.9 × 10^4^ CFU/mL to 1.0 × 10^4^ CFU/mL on average (n = 8)) (Fig. 3b). Thus, the tube capturing technique when used in infected rats could effectively remove pathogens suppressing pathogen concentration in the bloodstream. As previously blood clotting and clogging the tubes was not observed. CBC differential tests were performed to determine if the extracorporeal blood flow through the capturing tube can have any adverse effect in the hematological profile. No noticeable change in blood composition caused by extracorporeal capturing was not observed (detail analysis can be found in the supplementary information in section S2, Tables S3 and S4).

### Demonstration of high throughput operation

The capturing conditions described above can only handle relatively small sample volume because of the 0.5 mL/min flow rate. In order to process larger sample volumes, the flow rate must be increased to cover the entire sample volume within a given time frame. However, antibody-antigen binding may be hampered by an increase in flow rate. This was addressed by dividing the flow from one tube to four equivalent short tubes thereby reducing the flow rate in each individual tube. In order to demonstrate higher throughput capability, the sample volume was increased 4-fold (to 20 mL) and the flow rate was increased 10-fold (to 5 mL/min) (Fig. 2b). The flow rate in individual tubes was 1.25 mL/min. We observed negligible capturing in high flow rates of 5 mL/min flow without splitting. Increasing the flow rate up to 1.25 mL/min in individual tube did not appear to affect the capturing efficiency given that 96.5 ± 1.6% of the pathogens were captured compared to controls (Fig. 2c). This approach was also used to investigate the capturing small quantity of target pathogens in significantly larger sample volume as described in following sections. The experimental parameters, including sample volume, tube length, splitting, flow rate, circulation time, # cycles of circulations, antibody density on tube surface etc. were summarized and explained in supplementary information (Table S5 and section S4.1)

### Bacterial capturing in low concentrations and large sample volumes

**Figure 4:**
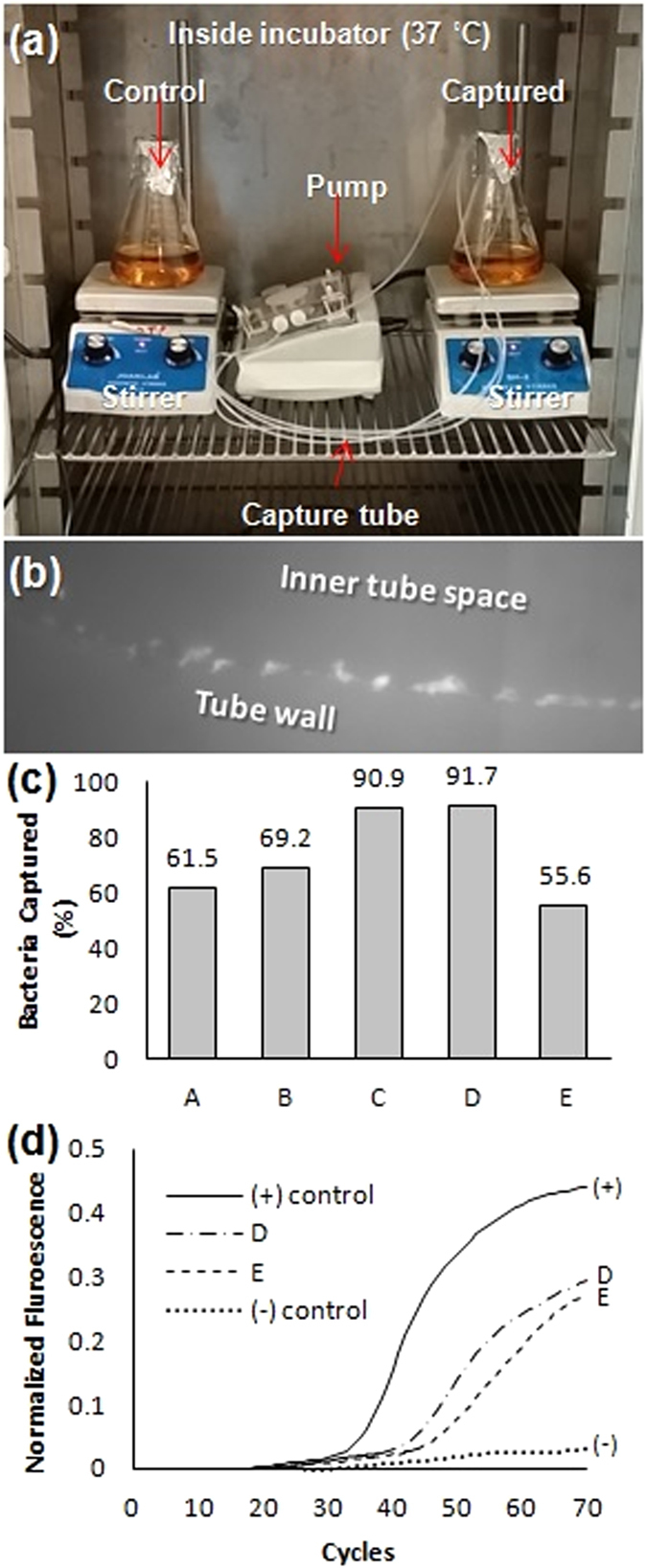
Tube capturing for very low concentration in large sample volume (**a**) Experiment set-up inside an incubator. (**b**) Fluorescence image of captured *S*. *typhimurium* on the tube inner walls stained by FITC-labeled primary *salmonella* antibody (cross sectional view). The stained bacteria were observed aligned on the surface of tube’s inner wall. (**c**) Capturing results under the following conditions: (A) 10^1^ CFU (10 CFU) in 100 mL, 7 hours of continuous flow, 120 cm tube. (B) 10^1^ CFU in 100 mL, 7 hours of continuous flow, 120 cm tube. (C) 10^1^ CFU in 100 mL, 6 hours of continuous flow, 120 cm tube. (D) 10° CFU (1 CFU) in 100 mL, 7 hours of continuous flow. (E) 10° CFU in 250 mL, 7 hours of continuous flow. (**d**) Positive RT PCR detection results from Condition D (DNA extraction by bead beating), and E (DNA extraction by commercial heating kit).

Capturing of bacteria in low concentrations and in large volumes of sample was investigated. Such conditions are encountered in food pathogen detection and environmental testing. Capturing low concentrations but in smaller volumes is relevant to bloodborne pathogen detection as well. *S*. *typhimurium* was inoculated into culture broth at the following initial concentrations, sample volumes, tube length, and total times: 10^1^ CFU in 100 mL with 120 cm (30 cm, 4-way split) tube for 7 hour capturing (n = 2, condition A, B) and 6 hour capturing (n = 1, condition C), 10^0^ CFU in 100 mL with 120 cm tube (30 cm, 4-way split) for 7 hour capturing (n = 1, condition D), and 10° CFU in 250 mL with 120 cm (30 cm, 4-way split) tube for 7 hour capturing (n = 1, condition E). The samples were circulated at a total flow rate of 6 mL/min (4 tubes were employed with a flow rate of 1.5 mL/min per individual tube) (Fig. 4a). After capturing, a short piece of tube was cut for fluorescence imaging and the rest of the tube was treated with lysis buffer for genomic DNA extraction for detection using real time PCR. Colony counting showed about 56–92% capture efficiency (Fig. 4c). Based on the capturing rate using colony counting, it was estimated that about 20,000–55,000 CFU of bacteria were captured inside the 1 mL volume tube. The presence of *S*. *typhimurium* at the concentration of 10° CFU in 100 mL (Condition D) and 250 mL (Condition E) were positively confirmed by RT PCR as shown in Fig. 4d, indicating ability to detect pathogens starting from low concentrations. The captured *S*. *typhimurium* was also confirmed by fluorescence microscopy imaging with FITC-labeled *Salmonella* antibody as a fluorescent tag (Fig. 4b). This result indicated that the target pathogen can be detected directly from tube by optical microscopy inspection.

**Figure 5:**
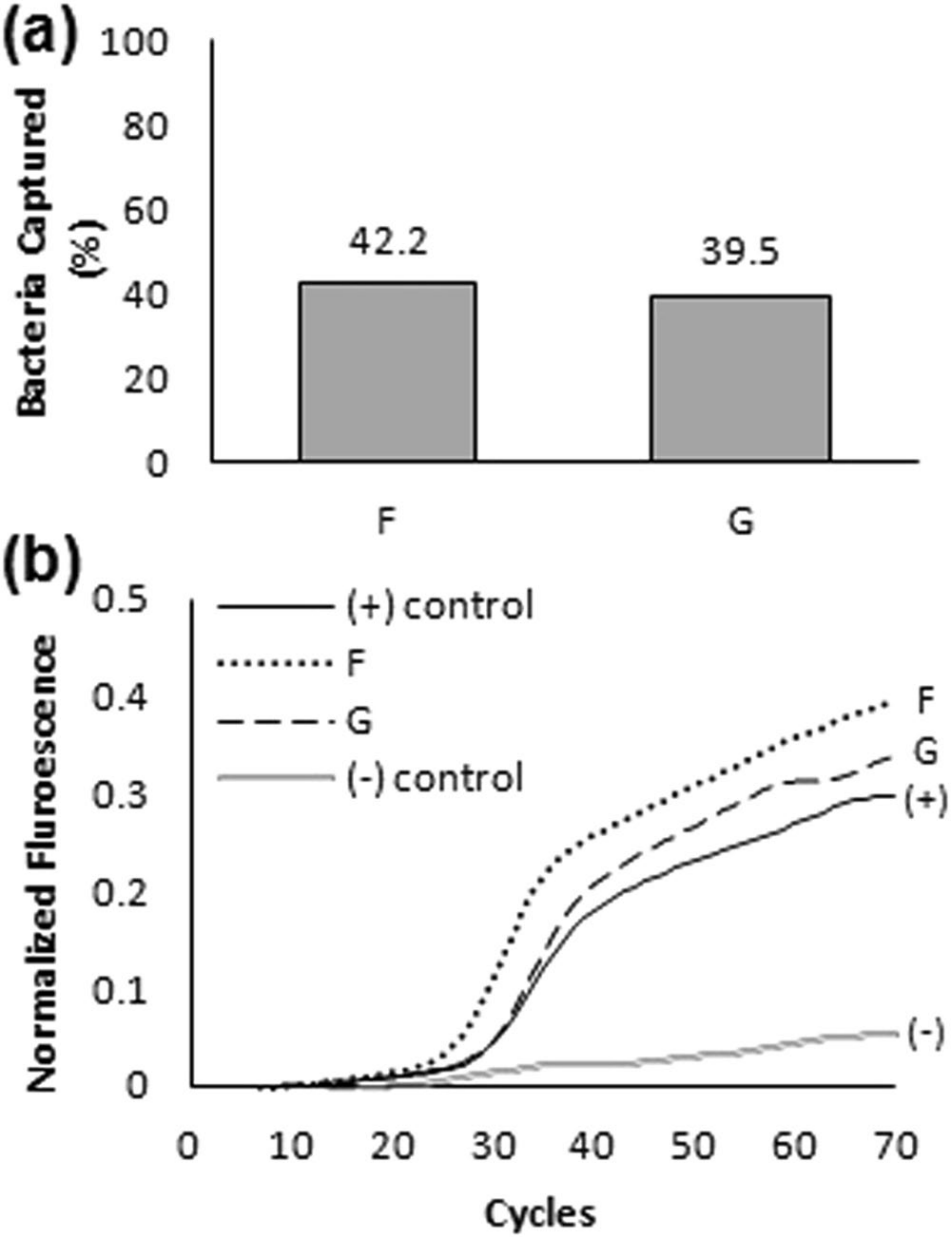
Effects of shorter tube length on tube capturing of very low concentration in large sample volume. (**a**) Capturing results under the following conditions: (F) 10^1^ CFU in 100 mL, 6 hours of continuous flow, 40 cm tube. (G) 10^1^ CFU in 100 mL, 6 hours of continuous flow, 40 cm tube. (**b**) Positive RT PCR detection results from Condition F and G (DNA extraction by heating in DI water).

Two conditions (Conditions F and G in Fig. 5) were added to examine whether the tube length can be shortened without reduction in detection efficiency. The major reason to reduce the total tube length from 120 cm (30 cm, 4-way split) to 40 cm (10 cm, 4-way split) was to examine if the same capturing efficiency per length can be achieved at a lower cost, given that for RT-PCR detection, 10 µL of DNA sample are needed and the rest of the sample is wasted. To demonstrate that this method works with other strains we used *S*. *typhimurium* strain (ATCC 14028). *S*. *typhimurium* at 10^1^ CFU in 100 mL tryptic soy broth (TSB) were circulated through shorter capturing tube (40 cm, 10 cm in a four-way split arrangement) for 6 hours. DNA was extracted directly from tube by inserting DI water in the tube and heating to 100 °C for 10 minutes. 10 µm from the content was then directly used for RT PCR without further purification steps. A reduction in capture efficiency to 40% for both Condition F and G was observed due to use of shorter tubes (Fig. 5a) However, DNA was extracted by heating without any additional purification steps (centrifugation, filtration, solvent exchange, magnetic separation, etc) minimizing sample loss and strong PCR amplification was obtained (Fig. 5b).

### Detachment of pathogen from tube

**Figure 6:**
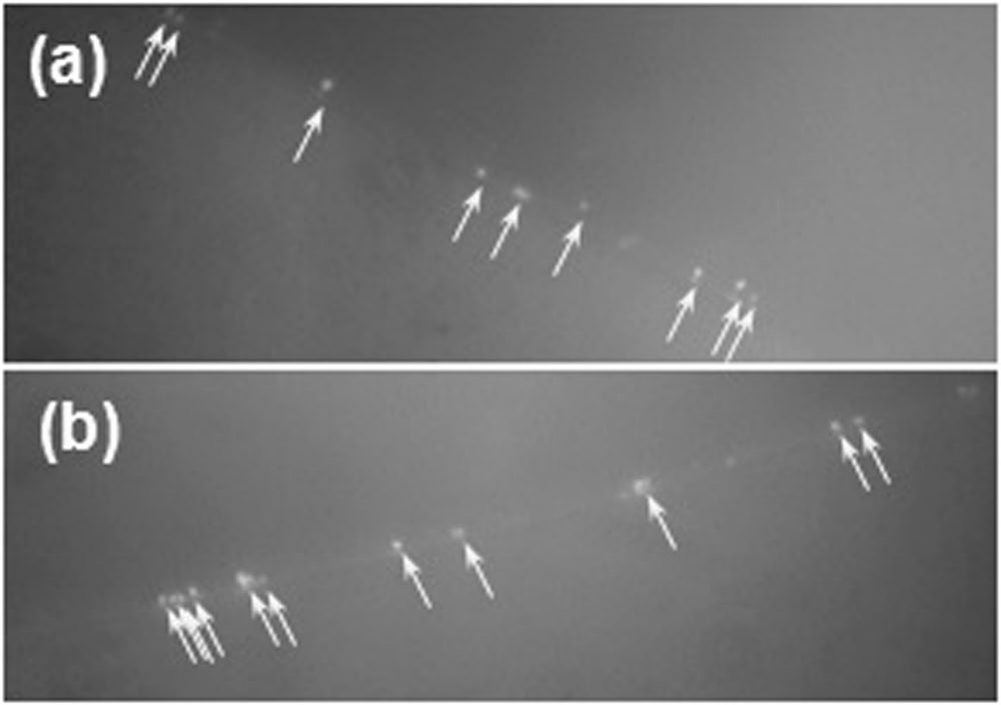
Confirmation of bacterial capturing by fluorescence imaging on detached bacteria (**a**) Fluorescence images of detached *S*. *aureus* by Pluriselect (neutral pH) detachment buffer, (**b**) Detached by IgG elution buffer from Thermo Fisher Scientific (pH 2.8), both stained with syto 64 dye. Bacteria were observed at the edge of the droplet (marked by arrows).

The remobilization of affinity captured pathogens is necessary for re-culturing and immunology based detection methods (ELISA and lateral flow immunoassays, etc.). Two commercially available chemicals, a detachment buffer from Pluriselect (neutral pH) and IgG elution buffer from Thermo Fisher Scientific (pH 2.8), were used. After incubation with either one of these two solutions, the detached bacteria were labeled with a fluorescent molecular probe, Syto 64. Remobilized bacteria were observed from both methods as shown in Fig. 6. Given that the initial bacterial input was low (10^1^ CFU/mL), bacteria were mostly found in the edge of the solution’s droplet (Fig. 6a,b). The detached bacteria were spread on agar plate. Only bacteria detached using the detachment buffer from Pluriselect resulted in growing colonies while no colonies were found when the acidic IgG elution buffer was used, indicating that the cells were damage by the acidic pH. The detachment buffer with neutral pH showed some bacterial re-growth, however the quantity of colonies was fewer compared to the number of bacteria observed in control groups. Recovery of detached bacteria using the neutral pH detachment buffer was confirmed with other pathogens such as *E*. *Coli* K-12 and *S*. *typhimurium*.

### Bacterial detection in a food matrix

The *S*. *typhimurium* study finds possible applications in food pathogen testing, because of the requirement to detect 1 bacterium (10^0^ CFU) in sample volumes of 250 mL according to government protocols (USDA Microbiology Laboratory Guidebook (MLG) 4.08 protocol and US FDA Bacteriological Analytical Manual (BAM) *Salmonella* protocol). In these type of tests turnaround times (i.e. time taken from sample preparation to positive detection) of one work shift (<8 hours) are highly desirable^21^. The detection of a small number of pathogens in <30 mL is also important in bloodborne infection detection^22^.

**Figure 7:**
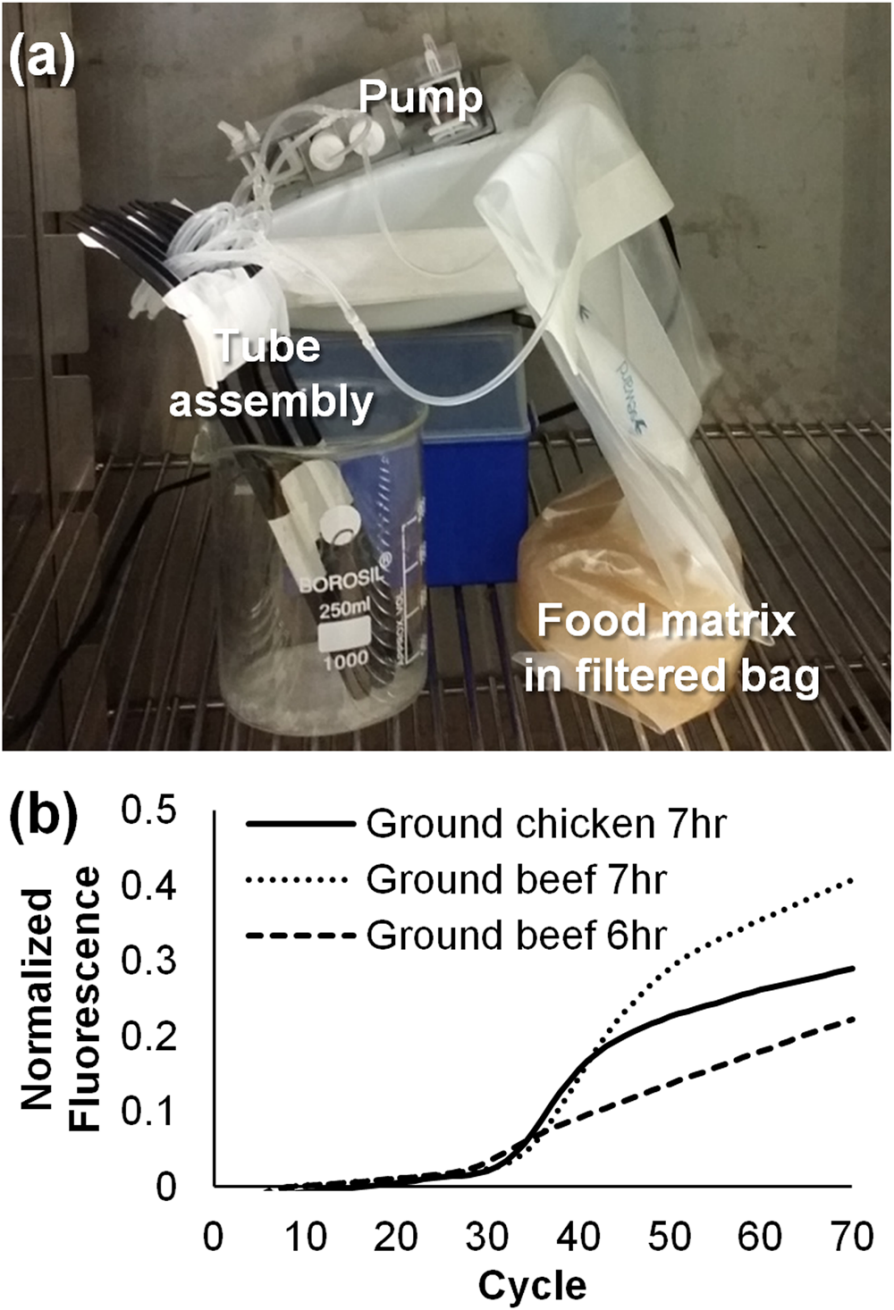
Tube capturing in a real food matrices; (**a**) Experiment set-up for capturing in ground chicken. (**b**) RT PCR detection of *S*. *typhimurium* in ground chicken at initial concentration ~25 CFU of *S*. *typhimurium* in 250 mL sample containing 25 g ground chicken in BPW and ground beef in Selective Romer Labs’ primary media with phage supplement at the same concentration, capturing completed within 6 or 7 hours (DNA extracted by magnetic bead method).

The applicability of this immuocapturing method to food pathogen detection was demonstrated using ground chicken as a food matrix at initial concentration of 25 CFU in 250 mL total sample volume (25 g of ground chicken in 225 ml of buffered peptone water (BPW, non-selective to *Salmonella* or 25 g of ground beef in 225 mL of Romer Labs Primary enrichment media supplemented with phage, selective to *Salmonella*) (Fig. 7a). Successful detection of *S*. *typhymurium* was confirmed after a 6 to 7-hour re-circulation process (Fig. 7b). These results demonstrate that using the tube as a concentration device detection can be achieved in a few hours vs. industry standard methods that require a minimum 18–24 hour of enrichment for detection as described in the BAM protocols. While, clogging has been a problem in microfluidic devices used for blood and food applications^23–25^, clogging was not observed when a 1 mm diameter tube was used in these studies in blood and food matrices (Tube concentration, detachment and detection of *S. typhimurium* from food matrix using lateral flow immunoassays﻿ is discussed in the supplement, *S3﻿*.).

## Conclusions

It has been demonstrated that this technique can effectively capture a wide range of bacterial quantities in blood and culture media *in vitro* and *in vivo*. Capturing was verified by colony counts on agar plates, by direct DNA extraction and subsequent real time PCR, by detachment and subsequent imaging and plating, and by directly imaging inside the tube. *In vitro* and *in vivo* results in constant flow, in low and high volume capturing, and faster flow rates were demonstrated.

## Methods

### Bacterial culture

Strains of *S*. *aureus*, MRSA and two *S*. *typhimurium* were purchased from the American Type Culture Collection (ATCC 12598, ATCC 43300, and ATCC 29630/14028 respectively). *S*. *aureus* and *S*. *typhimurium* (29630) were propagated in ATCC Medium 3 (nutrient broth or agar) at 37 °C. MRSA and *S. typhimurium* (14028) were expanded in tryptic soy broth (TSB) under the same conditions. Bacterial concentrations were determined by both OD-600 value (optical density at 600 nm wavelength), which was measured with a UV-VIS spectrometer (Spectronic 20 Genesys, Spectronic Instrument) in broth and its corresponding colony count from the agar plate. The initial OD-600 value was in the range of 0.02–0.04 (about 10^7^ CFU/mL) for *S*. *aureus* and MRSA, and about 0.01 (about 10^7^ CFU/mL) for *S*. *typhimurium*. MRSA was diluted further to make 10^4^ CFU/mL and *S*. *typhimurium* was diluted to 10^0^ CFU in 100–250 mL by serial dilution techniques from the enumerated high concentration^26^. Whole blood with an anticoagulant (sodium citrate) was purchased from commercial vendors such as Innovative Research (Novi, MI).

### Preparation of capturing tube

Polydimethylsiloxane (PDMS, Dow Corning Silastics) tubes with an internal diameter of 1.02 mm were used for this study. The tube length was 120 cm and tube was prepared as described in a previous publication^18^ with the minor modification of using different antibodies. *S*. *aureus* polyclonal antibody (PA1-7246, Life Technologies) was used for *S*. *aureus* capturing, penicillin binding protein 2 A (PBP2a) monoclonal antibody (10-P08B, Fitzgerald Industries International) was used for MRSA capturing, and BacTrace® Anti-Salmonella CSA-1 Antibody (01-91-99-MG, KPL) was used for *S*. *typhimurium* capturing. Following antibody immobilization, the tubes were stored at 4 °C until use.

### Bacterial capturing in blood

0.5 mL of bacterial suspension (10x concentrated from target concentration) were added to 4.5 mL of whole blood, resulting in 5 mL of 1x bacterial solution in blood (estimated bacterial input was about 10^7^ CFU/mL for *S*. *aureus* and about 10^4^ CFU/mL for MRSA) in sterile 15 mL culture tube. The initial bacterial concentration was estimated by monitoring the OD-600 value of sample diluted in the same way in broth instead of blood. Blood-bacteria mixtures were stirred with a mini magnetic stirrer to prevent blood from settling down by gravity over time. Antibody immobilized tubes were connected to the blood-bacteria mixture with both ends submerged into the solution (Fig. 2a).

The culture tubes containing bacteria spiked blood samples were placed in a water bath heated to 37 °C by a heating stirrer plate. The blood samples were agitated with a mini magnetic stirrer (7 mm x 2 mm) inside the solution. A 120-cm tube with antibody coating was connected to the blood sample in the water bath. The blood was circulated through the tube using a peristaltic pump (P-3, Pharmacia) at 0.5 mL/min for 5 hours (resulting in the entire 5 mL sample being recirculated about 30 times through the tube). The entire set up resided inside a biosafety cabinet. The experiments were repeated 5 times (n = 5). A duplicated control sample was run for each experiment to monitor the bacterial growth without capturing. In one case two experiments with MRSA were performed at the same time using one shared control.

### Demonstration of high throughput operation

In order to demonstrate high throughput, one tube was split into four capturing tubes with antibody coating using polypropylene Y-shaped barbed connectors (Warner Instruments, CT). 20 mL of the blood were spiked with MRSA (~10^4^ CFU/mL). A 5 mL/min flow rate using a high speed peristaltic pump (Cole-Parmer, IL) for 5 hours was used (20 mL sample was recirculated about 75 times through the tube). The sample collection for colony counting was performed as described previously (50 μL aliquots; collection times: 0, 1, 3, 5-hour time points; n = 4) (Fig. 3b).

### Processing samples and colony counting

Bacterial solution was pipetted from a culture tube and diluted into broth until the OD-600 value fell into the target range (OD-600: 0.02–0.04 roughly for 1 ~ 2 × 10^7^ CFU/mL of *S. aureus* and OD-600: and then 1000-fold further dilution in broth roughly for 10^4^ CFU/mL of MRSA). Each experiment was coupled with a duplicated control without circulation through tube, to account for the variation in bacterial concentration and growth patterns between batches (batch to batch variation).

The quantity of bacteria in the captured samples and their duplicated controls were determined by plating small aliquots of blood samples on agar plate and counting colonies on plates the following day. During the procedure, 50 μL blood samples were extracted at the following time intervals: 0 (before injection), 1 hour, 3 hours, and 5 hours (immediately after completion of the process). For *S. aureus*, the sample was diluted in 450 µL of nutrient broth (10-fold dilution) and diluted 3 additional times in same manner (100, 1000, 10000-fold). 10 μL of each of the diluted samples were streaked on a 5% sheep blood agar plate (Fisher Scientific) that was divided into quadrants. For MRSA, 50 μL blood samples were directly spread on the entire agar plates at 0, 1, and 3-hour time points. Samples at 5-hour time point were 100-fold diluted in broth (to avoid an overly crowded plate) and then spread on agar plates. The bacteria were allowed to grow overnight at 37 °C. The number of colonies was then counted using the particle analysis function in ImageJ (National Institutes of Health) and CFU/mL values were calculated by applying all the dilution factors. The difference from the control and the test sample provided an estimate of the bacteria removed from sample by the tube. Bacterial capturing was analyzed and presented as a percentage reduction compared to controls (mean ± standard error of mean (SEM)) for the 5-hour processes. SEM is defined as: SEM = standard deviation/(square root of n-1). For comparison purposes, percentages were used because of the batch-to-batch variations in bacterial growth rate that were observed in controls (the full sets of data are presented in the supplementary information, Fig. S1, Tables S1 and S2).

### Establishing of *S*. *aureus* infections in rats

All animal protocols were reviewed and approved by the Institutional Animal Care and Use Committee (approval #: IACUC 15-007 CR01) of the Florida International University and all experiments were performed in accordance with relevant guidelines and regulations. 12-week old Wistar rats (with body weight around 350 g) were obtained from a commercial vendor (Envigo, IN). 2 mL of 10^10^ CFU/mL *S*. *aureus* suspension in PBS were administered via an intraperitoneal (IP) injection under animal biosafety level (ABSL)-2 conditions. Blood samples were collected at 3, 4, 6, 8 hours following the injection. 100 µL of blood samples were spread on sheep blood agar plates at each time point. The bacterial concentrations in blood were determined by colony counting the following day.

### In *vivo* capturing in *S*. *aureus* in infected rats

The rats were purchased with surgically installed catheter implantations in the jugular vein and the carotid artery from a commercial vendor (Envigo, IN). Blood samples were collected just before injection. The rats were infected with *S*. *aureus* (2 mL of 10^10^ CFU/mL) similarly to the controls. A 3-hour waiting period was established to allow the bacteria to invade the blood stream. Following the 3-hour waiting period, two *S*. *aureus* antibody immobilized tubes were connected in series (yielding an overall length of 240 mm for each rat) and extracorporeal circulation of blood was initiated at 0.5 mL/min flow rate. The blood was circulated for 5 hours through the tubes (estimated using 7% of body weight (BW) for blood volume (BV), therefore assuming that the rats possess 25 mL total blood volume, the entire blood was recirculated 6 cycles through the tube). Blood was drawn at the following time points after the start of the procedure: 0, 1, 3, 5 hours. 100 µL samples were spread on 5% sheep blood agar plate for overnight culturing. The procedure is depicted in Fig. 3a.

### Bacterial capturing in low concentrations in large sample volumes

*S*. *typhimurium* was used for capturing in low concentration studies. 1 mL of bacterial suspension that was prepared by serial dilution from enumerated higher concentration. This was mixed with 99 mL and 249 mL of broth in Erlenmeyer flasks (tested initial concentrations were 10^1^ CFU in 100 mL for 7 hour capturing (n = 2, condition A, B) and 6 hours capturing (n = 1, condition C), 10^0^ CFU in 100 mL for 7 hour capturing (n = 1, condition D), and 10^0^ CFU in 250 mL for 7 hour capturing (n = 1, condition E)). The entire experiment was performed inside an incubator at 37 °C (Fig. 4a). To handle larger volume, the 120 cm *S*. *typhimurium* antibody coated tube was cut in four 30-cm length parts that were connected in parallel using a y-shaped connector. The 4-way assembly was connected with an unmodified tube which was connected to a high speed peristaltic pump (Cole-Parmer, IL). The flow rate was adjusted to ~6 mL/min (1.5 mL/min per individual tube). The bacterial growth was monitored by spreading 100 µL aliquot from the capturing and control flasks onto the agar plate, every hour until the end of the experiment. It is estimated that the 250 mL sample was circulated about 10 times through the tube in 7 hours, about 21 time for the 100 mL sample during the 6-hour experiment, and 25 times for the 100 mL sample during the 7-hour experiment. Two additional conditions (condition F and G) with S. typhimurium (ATCC 14028 strain) using shorter tube lengths (40 cm) were performed at a starting concentration of 10^1^ CFU in 100 mL for 6 hours capturing at 42 °C (42 °C was used to promote bacterial growth^27–31^). DNA was extracted directly from the tube by heating the tube while filled with DI water at 100 °C for 10 minutes without further purification steps. Capturing was verified by plating and PCR detection in pure culture..

### Bacterial detection in a food matrix

The capturing performance was confirmed using ground chicken and ground beef as food matrices (Fig. 7a). Ground meat was processed as described in the Bacteriological Analytical Manual (BAM) protocol (Chapter 5. C. 15) with slight modifications. 25 g of ground meats were added into a Seward filtered sample bag. Then 225 mL of BPW (for ground chicken) or a selective media (Romer Labs Primary enrichment media supplemented with phage, for ground beef) was added into the bag. The sample was manually blended for 5 minutes. *S*. *typhimurium* was inoculated so that the starting concentration would be about 25 CFU. In the first tests the solution was incubated for 7 hours at 35 **°**C (later 42 °C also attempted)^27–31^. The tube was connected to the filter bag inside the incubator at the beginning of the incubation. The optimized experimental parameters for capturing in blood, pure culture, and food matrix were summarized in the supplementary information section (Table S5).

### Confirmation of bacterial capturing on tube surface by fluorescence imaging

Following the capturing process, about 5 cm were cut from tube assembly and used for fluorescence imaging. The remaining of tube was processed for DNA extraction and subsequent PCR detection described below. The tube was further cut with a sharp razor blade in small pieces (1–2 mm long). The pieces were submerged in PBS buffer (1.5 mL). Then, 20 µL of FITC-labeled *Salmonella* antibody (KPL) was added into the solution. After 30 minutes of staining, the antibody solution was removed and the tubes were gently washed with fresh PBS. Then, the inner cross sectional surface of tube was examined under a microscope.

### Detection *S*. *typhimurium* with real time PCR

Genomic DNA from captured *S*. *typhimurium* were directly extracted from tube. Three extraction techniques were employed following manufacturer instructions: heating extraction using mericon DNA bacteria kit (Qiagen) was used in Condition E, a magnetic extraction method using Dynabeads DNA direct universal kit (Thermo Fisher Scientific) was used in Condition D and ground chicken tests. DNA extraction was also done by heating at 100 °C in DI water for 10 minutes. Real time PCR was performed using the mericon *Salmonella spp*. kit (Qiagen). 10 µL of extracted DNA sample was mixed with 10 µL of mastermix (reconstituted mericon assay). A positive control with 10 µL purified *salmonella* DNA (provided with the kit) and a negative control with 10 µL dilution buffer (no DNA) were prepared as well. The PCR reactions were performed using a Rotor-gene-Q (Qiagen) RT PCR instrument. The sample was heated to 95 °C for 5 min. Then, up to 70 cycles were performed (each cycle included denaturation at 95 °C for 15 sec, annealing at 60 °C for 15 sec, and extension at 72 °C for 10 sec). The amplification was determined by monitoring the fluorescence of the FAM (495/520 nm excitation/emission) probe for the target gene and of the MAX (524/557 nm excitation/emission) probe for internal control.

### Detachment of pathogen from tube

The captured bacteria were detached using two different detachment solutions: a Detachment buffer from Pluriselect (neutral pH) and an antigen-antibody dissociation buffer (IgG elution buffer) from Thermo Fisher Scientific (pH 2.8). Initially, a test was performed using 30 cm tubes. Two antibody coated tubes were filled with 200 µL of 10^1^ CFU/mL *S*. *aureus* in broth and incubated at 37 °C for 5 hours. A 1 mL of 10^1^ CFU/mL *S*. *aureus* solution was incubated for control. After 5 hours, the bacterial solution was carefully removed from the tube and collected. 100 µL of the collected residual solution along with the control solution were spread on agar plates for colony counting the next day. Emptied tubes were filled with the Pluriselect detachment buffer or the IgG Elution buffer for 15 minutes. The solutions were collected and centrifuged to pellet the detached bacteria at 3000 RPM for 5 minutes. Supernatants were carefully removed and bacterial pellets were resuspended in 20 µL PBS. 1 µL of syto 64 staining dye (599/619 nm, excitation/emission, Thermo Fisher Scientific) was added into each suspension. After 15 minutes, the cell suspension was spun down and remaining supernatant was removed as described before. The bacterial pellet was resuspended in 50 µL PBS and imaged on fluorescent microscopy. After imaging, the bacterial solution was recovered and the entire solution was spread on agar plates for colony counting.

### Data availability statement

This report does not include any data for mandatory deposition and full data sets were presented in the supplementary information.

## Electronic supplementary material

Supplementary information

## References

[1] Noble RT, Weisberg SB (2005). A review of technologies for rapid detection of bacteria in recreational waters. Journal of water and health.

[2] Swaminathan B, Feng P (1994). Rapid detection of food-borne pathogenic bacteria. Annual Reviews in Microbiology.

[3] Mairhofer J, Roppert K, Ertl P (2009). Microfluidic systems for pathogen sensing: a review. Sensors.

[4] Inci, F. *et al*. Portable microfluidic chip for detection of Escherichia coli in produce and blood. (2012). 10.2147/IJN.S29629 PMC3368510 22679370

[5] Peng Z-Y (2013). Development of venovenous extracorporeal blood purification circuits in rodents for sepsis. Journal of Surgical Research.

[6] Saucedo-Zeni N (2012). A novel method for the *in vivo* isolation of circulating tumor cells from peripheral blood of cancer patients using a functionalized and structured medical wire. International journal of oncology.

[7] Zhang H (2015). *In vivo* capture of circulating tumor cells based on transfusion with a vein indwelling needle. ACS applied materials & interfaces.

[8] Prentice N, Murray JS, Scott MF, Coombs JP, Parton A (2006). Rapid isolation and detection of Escherichia coli O157: H7 in fresh produce. Journal of Rapid Methods & Automation in Microbiology.

[9] Beyor N, Seo TS, Liu P, Mathies RA (2008). Immunomagnetic bead-based cell concentration microdevice for dilute pathogen detection. Biomedical microdevices.

[10] Sanchis A (2007). Dielectric characterization of bacterial cells using dielectrophoresis. Bioelectromagnetics.

[11] Fedio WM (2011). Detection of E. coli O157: H7 in raw ground beef by Pathatrix™ immunomagnetic-separation, real-time PCR and cultural methods. International journal of food microbiology.

[12] Didar TF (2015). Improved treatment of systemic blood infections using antibiotics with extracorporeal opsonin hemoadsorption. Biomaterials.

[13] Kang, J. H. *et al*. An extracorporeal blood-cleansing device for sepsis therapy. *Nature medicine* (2014). 10.1038/nm.3640 25216635

[14] Mattsby-Baltzer I (2011). Affinity Apheresis for Treatment of Bacteremia caused by Staphylococcus aureus and/or Methicillin-Resistant S. aureus (MRSA). J Microbiol Biotechnol.

[15] McCrea K, Ward R, LaRosa SP (2014). Removal of Carbapenem-Resistant Enterobacteriaceae (CRE) from blood by heparin-functional hemoperfusion media. PloS one.

[16] Shoji H (2003). Extracorporeal endotoxin removal for the treatment of sepsis: endotoxin adsorption cartridge (Toraymyxin). Therapeutic Apheresis and Dialysis.

[17] Zhang J (2015). Effects of Hemoadsorption with a Novel Adsorbent on Sepsis: *In vivo* and *in vitro* Study. Blood purification.

[18] Gaitas A, Kim G (2015). Chemically modified plastic tube for high volume removal and collection of circulating tumor cells. PloS one.

[19] Cross AS, Opal S, Sadoff J, Gemski P (1993). Choice of bacteria in animal models of sepsis. Infection and immunity.

[20] St Geme JW, Bell LM, Baumgart S, D’Angio CT, Harris MC (1990). Distinguishing sepsis from blood culture contamination in young infants with blood cultures growing coagulase-negative staphylococci. Pediatrics.

[21] Bhunia AK (2014). One day to one hour: how quickly can foodborne pathogens be detected?. Future microbiology.

[22] Dark PM, Dean P, Warhurst G (2009). Bench-to-bedside review: the promise of rapid infection diagnosis during sepsis using polymerase chain reaction-based pathogen detection. Critical care.

[23] VanDelinder V, Groisman A (2006). Separation of plasma from whole human blood in a continuous cross-flow in a molded microfluidic device. Analytical chemistry.

[24] He L, Deen BD, Pagel AH, Diez-Gonzalez F, Labuza TP (2013). Concentration, detection and discrimination of Bacillus anthracis spores in orange juice using aptamer based surface enhanced Raman spectroscopy. Analyst.

[25] Heo J, Hua SZ (2009). An overview of recent strategies in pathogen sensing. Sensors.

[26] Garthright, W. Most Probable Number From Serial Dilutions. FDA Bacteriological Analytical Manual, Appendix 2. *Food and Drug Administration*, *Washington* (1995).

[27] Davies P (2000). Comparison of methods for isolating Salmonella bacteria from faeces of naturally infected pigs. Journal of applied microbiology.

[28] Nabbut NH (1973). Elevated temperature technique for the isolation of salmonellas from sewage and human faeces. J Hyg (Lond).

[29] Romer L. *RapidChek® SELECT™ for Salmonella*, http://www.medica-tec.com/arg/files/SDI-Rapidchek%20Salmonela%20SELECT-Folleto.pdf.

[30] Thermo Fisher Scientific. *Salmonella Rapid Culture Method Using ONE Broth Salmonella and Brilliance™ Salmonella*, https://tools.thermofisher.com/content/sfs/brochures/AOAC-RI-Certificate-Salmonella-Precis-Method.pdf (2016).

[31] Neogen F Safety. *RAPPAPORT-VASSILIADIS SALMONELLA ENRICHMENT BROTH* (*7730*), http://foodsafety.neogen.com/pdf/acumedia_pi/7730_pi.pdf (2014).

