# Supplementary material for: A Novel Pathogen Capturing Device for Removal and Detection
Source: Sci Rep. 2017 Jul 17;7:5552. doi: 10.1038/s41598-017-05854-4 (PMC5514083; doi:10.1038/s41598-017-05854-4)
Supplement: Supplementary file 1 — Supplementary information [file 41598_2017_5854_MOESM1_ESM.pdf]

Supplementary Information:

A Novel Pathogen Capturing Device for Removal and Detection

Gwangseong Kim[1, 2], Horatiu Vinerean[3], and Angelo Gaitas[1, 2, *]

[1] Kytaro, Inc., Miami, FL, USA 33199

[2] Department of Electrical and Computer Engineering, Florida International University, Miami, Florida, USA 33199

[3] Office of Research and Economic Development, The Office of Laboratory Animal Research, Florida International University, Miami, Florida, USA 33199

[*] Corresponding authors: agaitas@fiu.edu (A.G.)

11200 SW 8th Street, MARC 430

Miami, FL 33199

(734) 913-2608 - office

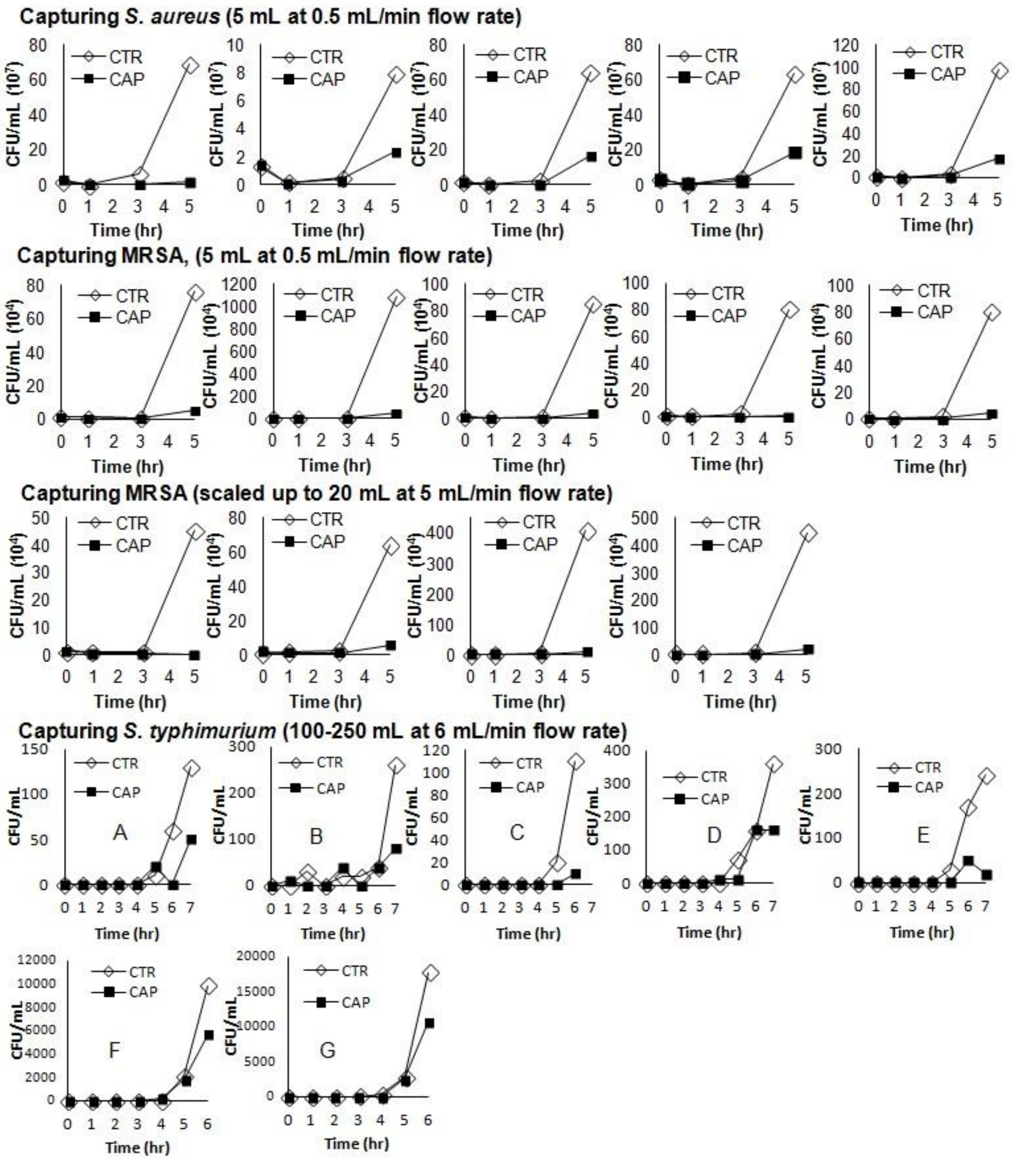

Figure S1. Full record of bacterial growth profile analyzed by colony counting for: *S. aureus* capturing, MRSA capturing, MRSA capturing in high throughput condition, and *S. typhimurium* capturing.

CFU/mL	*S. aureus*				MRSA			
	Tube capturing		Control		Tube capturing		Control	
	Initial	5 hour	Initial	5 hour	Initial	5 hour	Initial	5 hour
Experiment 1	2.71×10^7	1.70×10^7	1.81×10^7	6.90×10^8	1.87×10^4	5.66×10^4	1.74×10^4	7.61×10^5
Experiment 2	1.47×10^7	2.39×10^7	1.27×10^7	7.88×10^7	1.84×10^4	4.76×10^5	2.00×10^4	1.08×10^7
Experiment 3	1.69×10^7	1.65×10^8	1.61×10^7	6.42×10^8	8.66×10^3	4.00×10^4	8.88×10^3	8.52×10^5
Experiment 4	1.97×10^7	1.79×10^8	1.74×10^7	9.77×10^8	8.72×10^3	8.00×10^3	1.11×10^4	8.00×10^5
Experiment 5	3.21×10^7	1.84×10^8	3.32×10^7	6.36×10^8	1.32×10^4	5.16×10^4	1.11×10^4	8.00×10^5

Table S1. Bacterial concentration (CFU/mL) in the sample before and after the 5-hour process in 5 mL sample volume and 0.5 mL flow rates. Growth is suppressed using tube capturing. Experiments 4 and 5 in MRSA were performed together with a shared control.

Scale up MRSA CFU/mL	Tube capturing		Control	
	Initial	5 hour	Initial	5 hour
Trial 1	1.33×10^4	2.00×10^3	1.33×10^4	4.52×10^5
Trial 2	1.59×10^4	5.40×10^4	1.32×10^4	6.42×10^5
Trial 3	1.65×10^4	9.80×10^4	1.56×10^4	4.07×10^6
Trial 4	1.35×10^4	2.22×10^5	1.18×10^4	4.45×10^6

Table S2. Bacterial concentration (CFU/mL) in the sample before and after by 5-hour of 20 mL sample volume at 5 mL flow rates.

S1.1. Preliminary confirmation - PCR detection of MRSA from blood

The capturing of bacteria by the antibody conjugated tube and subsequent lysis and detection by RT-PCR were confirmed in MRSA spiked sheep blood. 0.5 mL of 10 CFU/mL serial diluted MRSA (strain 43300) were spiked into 4.5 mL sheep blood (purchased from Hemostat Laboratories, Dixon, CA) supplemented with Na-citrate anticoagulant and pure tryptic soy broth (TSB), making 5 mL solution containing 5 CFU of MRSA. A 120 cm PBP2a antibody coated tube was connected to a vial containing the blood. The blood was circulated for 5 hours at 37 ˚C. Then, the tube was rinsed with 10 mL DI water. MRSA DNA was extracted from the tube by heating the tube filled with DI water to 100 ˚C for 10 min. RT-PCR was run with Qiagen mericon S. aureus kit. 10 µL of the extracted DNA sample was mixed with 10 µL of mastermix (reconstituted mericon assay). The sample was heated to 95 ˚C. Then, up to 70 cycles were performed (each cycle included denaturation at 95 ˚C for 15 sec, annealing at 60 ˚C for 15 sec, and extension at 72 ˚C for 10 sec). As shown in supplementary Fig S2, the capture of MRSA in the tube was positively confirmed by RT-PCR.

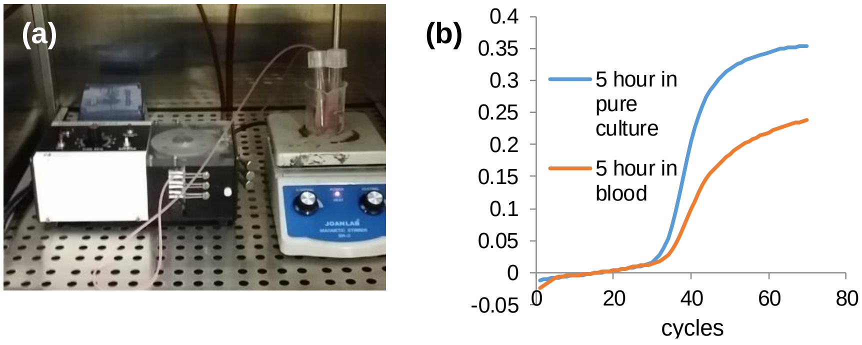

Figure S2. Positive detection of MRSA from capturing tube confirmed by RT-PCR (a) experimental set-up (b) PCR results after 5 hours of capturing inside the tube for blood and pure media samples.

S2.1. Method: Blood analysis by complete blood counting with differential test

During *in vivo* studies, about 1 mL of rat blood samples were collected into vacutainer tubes with EDTA at the following time points: before injection of bacteria and at the end of 8 hours after infection (with and without tube capturing). The blood samples were shipped to a commercial veterinary diagnostics company (Antech Diagnostics, TN) for complete blood count with differential (CBC Differential) analysis. CBC differential analysis was performed for 8 rats: 4 control and 4 that underwent the tube capturing process. The results were statistically analyzed using T-test (two tailed, not paired) between the values before infection and the values 8 hours after infection for the group that underwent tube capturing and the control group. Capturing was initiated 3 hours after infection and the process lasted for 5 hours. By comparing the blood results before infection and 8 hours post infection, the impact of the infection can be assessed. By comparing the group that underwent tube capturing vs the control group we can assess if the tube capturing process affected the rats. Statistically significant results are identified and discussed. The procedures are summarized in the diagram below.

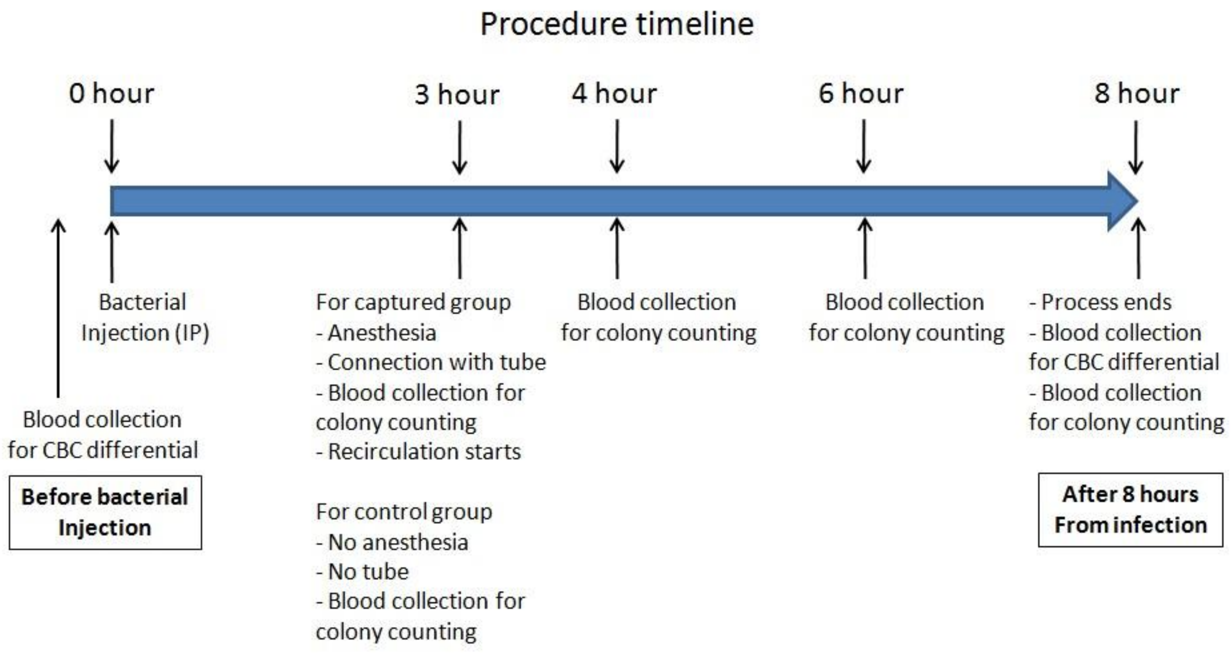

S2.2. Result: CBC differential analysis

The results are summarized in supplementary table S3 and S4. Blood samples from infected and cannulated rats (n=4) that underwent the capturing process were compared to control groups of infected rats (n=4) without cannulation and not treated by capturing process. Both groups showed similarly significant reduction in white blood cell (WBC) count. However, this reduction was observed from both captured and control groups without any statistically significant difference. The reduction in WBC count may be attributed to the effect of bacterial infection. Significantly elevated or decreased WBC count can be caused by sepsis[1,2], indicating the rats were in a septic condition. Differential analysis exhibited an elevation in neutrophil (acting against bacterial infection) count ratio from ~ 12 % to ~ 45 % in captured and from ~ 11 % to ~ 40 % in control and the decline in lymphocyte (acting against viral infection) count ratio from ~ 86 % to ~ 50 % in the captured and from ~ 86 % to ~ 45 % in the control. This trend is an indication of bacterial infection [3,4]. Red blood cell (RBC) count in the treated rats showed slight reduction after capturing, while RBC count in controls showed slight increase. This effect was also confirmed significant in the captured vs. control comparison. The reduction in RBC count is due to either temporary blood loss because of the extracorporeal circulation or due to minor dilution induced by the heparin injection (about 1 mL over 5 hours) to prevent potential clotting in the catheter and tube (only the captured group needed heparin, note that the total blood volume of rats is about 25 mL). However, this effect was still tolerable because the overall RBC counts are within the reference range (provided by Antech Diagnostics). Hemoglobin level and hematocrit value in the before bacterial injection captured group vs. before bacterial injection control group showed a meaningful difference. This difference was observed in healthy rats before infection, and might be due to the implanted catheter. Reduction in hemoglobin

concentration and hematocrit in the rats that underwent capturing can be attributed to the Heparin injection. Controls showed statistically significant increase in mean corpuscular volume (MCV) and increase in mean corpuscular hemoglobin concentration (MCHC). These changes were not observed in the captured group. However, comparison between the captured vs. control groups showed that these two changes were not statistically significant.

CBC	Captured		Control		
Components unit	Before bacterial injection	8 hours after infection	Before bacterial injection	8 hours after infection	Reference Range
WBC 10^3/uL	9.88 ± 1.23	4.10 ± 2.47	11.35 ± 0.94	3.55 ± 0.46	5.50 - 11.0 *
RBC 10^6/uL	8.03 ± 0.24	7.18 ± 0.12	8.53 ± 0.23	9.00 ± 0.52	5.5 - 10.5 *
Hemoglobin g/dL	14.18 ± 0.41	12.90 ± 0.41	15.73 ± 0.29	16.48 ± 0.86	14.7 ± 0.7 $
Hematocrit %	49.25 ± 1.44	43.00 ± 1.56	54.50 ± 1.11	54.00 ± 2.71	33.0 - 55.0 *
MCV fL	61.50 ± 2.03	60.00 ± 1.83	64.00 ± 1.25	60.00 ± 1.25	60.9 ± 1.6 $
MCH pg	17.68 ± 0.50	17.98 ± 0.46	18.45 ± 0.19	18.33 ± 0.17	19.4 ± 0.5 $
MCHC g/dL	28.78 ± 0.25	30.08 ± 1.16	28.85 ± 0.28	30.50 ± 0.44	31.8 ± 0.3 $
Platelet count 10^3/uL	619.50 ± 96.47	618.00 ± 169.11	928.75 ± 198.57	869.00 ± 77.56	
Differential					
Neutrophils /uL (%)	1215.25 ± 170.68 (12.50 ± 1.67)	2389 ± 1951.551 (45.00 ± 11.40)	1224.50 ± 113.98 (11.00 ± 1.49)	1452.50 ± 348.54 (40.50 ± 7.31)	(9.3 ± 4.5) $
Bands /uL (%)	0.00 ± 0.00 (0.00 ± 0.00)	0.00 ± 0.00 (0.00 ± 0.00)	0.00 ± 0.00 (0.00 ± 0.00)	0.00 ± 0.00 (0.00 ± 0.00)	
Lymphocytes /uL (%)	8474.50 ± 1070.32 (85.75 ± 0.99)	1568.00 ± 506.52 (50.00 ± 9.82)	9743.00 ± 861.93 (85.75 ± 0.73)	1544.25 ± 118.13 (44.50 ± 4.18)	(81.5 ± 5.3) $
Monocytes /uL (%)	156.25 ± 71.91 (1.5 ± 0.75)	137.75 ± 41.63 (4.75 ± 4.72)	352.50 ± 198.06 (3.00 ± 1.49)	538.75 ± 248.95 (14.5 ± 5.47)	(9.0 ± 2.6) $
Eosinophils /uL (%)	29.00 ± 33.49 (0.25 ± 0.29)	5.25 ± 6.06 (0.25 ± 0.29)	0.00 ± 0.00 (0.00 ± 0.00)	7.50 ± 8.66 (0.25 ± 0.29)	(0.1 ± 0.1) $
Basophils /uL (%)	0.00 ± 0.00 (0.00 ± 0.00)	0.00 ± 0.00 (0.00 ± 0.00)	30.00 ± 34.64 (0.25 ± 0.29)	0.00 ± 0.00 (0.00 ± 0.00)	(0.1 ± 0.1) $

*: provided by Antech diagnostics, $: provided by Envigo

Table S3. Complete Blood Count with Differential results summary.

T-test p	Before bacterial injection vs.	Tube caputing vs. control

values	8 hours after infection			
based on count	Captured	Control	Before-bacterial injection	8 hours after infection
WBC	0.05190	**0.00014**	0.31433	0.80855
RBC	**0.01085**	0.37637	0.13858	**0.00785**
hemoglobin	**0.04368**	0.37687	**0.01193**	**0.00488**
hematocrit	**0.01465**	0.85004	**0.01572**	**0.00664**
MCV	0.54898	**0.03966**	0.27083	1.00000
MCH	0.62756	0.59563	0.14551	0.44045
MCHC	0.25114	**0.01126**	0.82583	0.70566
Platelet count	0.99319	0.75719	0.15689	0.17028
Differential Based on %				
Neutrophils	**0.01732**	**0.00383**	0.46799	0.71447
Bands				
Lymphocytes	0.00580	**0.00003**	1.00000	0.57352
Monocytes	0.09268	0.05758	0.33875	0.09716
Eosinophils	1.00000	0.35592	0.35592	1.00000
Basophils			0.35592	

Table S4. Statistical analysis of CBC differential results, presented by p values from T-test. Bold values indicated statistically significant difference (p value less than 0.05).

References (for Supplementary Information)

1 Chen, W. L. & Kuo, C. D. Characteristics of heart rate variability can predict impending septic shock in emergency department patients with sepsis. *Academic emergency medicine* 14, 392-397 (2007).

2 Rivers, E. *et al.* Early goal-directed therapy in the treatment of severe sepsis and septic shock. *New England Journal of Medicine* 345, 1368-1377 (2001).

3 de Jager, C. P. *et al.* Lymphocytopenia and neutrophil-lymphocyte count ratio predict bacteremia better than conventional infection markers in an emergency care unit. *Critical care* 14, 1 (2010).

4 Naess, A., Nilssen, S. S., Mo, R., Eide, G. E. & Sjursen, H. Role of neutrophil to lymphocyte and monocyte to lymphocyte ratios in the diagnosis of bacterial infection in patients with fever. *Infection*, 1-9, doi:10.1007/s15010-016-0972-1 (2016).

S3.1. Method: Expedited detection of *S. typhimurium* from food matrix using lateral flow immunoassay

The capturing performance in ground chicken was also confirmed using two lateral flow immunoassay devices, namely Neogen’s Reveal 2.0 *Salmonella* strip and Romer Labs' Rapidchek SELECT *Salmonella* test strip. Lateral flow immunoassays are low cost, simple, easy to use, and portable (point-of-care) detection methods, but they require considerably longer enrichment time than PCR because of a higher limit of detection (LOD, ~ 10^6 CFU/mL). Ground chicken was prepared as described in the previous section. 25 g of ground chicken were added into a Seward filtered sample bag. 225 mL of tryptic soy broth (TSB, non-selective) were added into the bag making the final total volume ~ 250 mL. The sample was blended manually for 5 minutes. About 25 CFU of *S. typhimurium* (ATCC 14028) were inoculated in the 250 mL. In the first test the solution was incubated for 14 hours at 42 ˚C. The length of tube was reduced to 40 cm (in a 4-way split). The tube was connected to the filter bag inside the incubator and the sample was circulated at a 6 mL/min flow rate. After 14 hours of capturing, the tube was gently rinsed with DI water and then filled with 1 mL of the Pluriselect detachment buffer solution. After a 15-min detachment, the solution was collected and centrifuged at 3000 RPM for 5 minutes. The supernatant was carefully removed not to interrupt the pellet. The pellet was resuspended in 1 mL fresh TSB and then centrifuged again at 3000 RPM. After careful removal of supernatant, the pellet was resuspended in 200 µL TSB. Then, a lateral flow strip (Neogen's Reveal 2.0 *Salmonella* strip and Romer Labs' RapidChek SELECT *Salmonella* strip) was inserted into the suspension for 15 minutes. An experiment was repeated using Romer Labs Primary enrichment media (225 mL) with primary media supplement containing phage to

suppress the growth of other bacteria. The other parameters remained the same. However, the enrichment time was reduced from 14 hour to 9 hours.

S3.2. Result: Expedited detection by lateral flow immunoassay

S. typhimurium (ATCC 14028) starting from 25 CFU in 250 mL ground chicken sample (25 g) could be detected within 14 hours using two commercially available lateral flow immunoassay devices (Neogen Reveal 2.0 *Salmonella* strip and Romer Labs RapidChek SELECT *Salmonella* strip) as shown in supplementary Figure S2. According to manufacturers' protocols, Neogen Reveal 2.0 *Salmonella* strip requires 20-24 hours of enrichment time in selective RV medium until lateral flow test. Romer Labs RapidChek SELECT *Salmonella* strip requires 16-22 hours of primary enrichment in a selective primary media and then another round of 16-22 hours of secondary enrichment in a selective secondary media until lateral flow test (overall 36-44 hours). Therefore, our positive detection at 14 hours demonstrated that this immunocapturing technique could considerably expedited the detection of target pathogen by lateral flow immunoassays. It should be noted that positive detection at 14 hours was obtained from non-selective media, thus the growth of *S. typhimurium* is be slower than the selective media used in lateral flow assay protocols (which suppress other organisms while favor the target organism's growth). This happens because in the non-selective media the nutrients are shared with other organisms (natural flora). Thus, we repeated the experiment with selective media, Romer Labs Primary enrichment media with phage supplement, and confirmed that enough bacteria can be concentrated for positive detection in 9 hours.

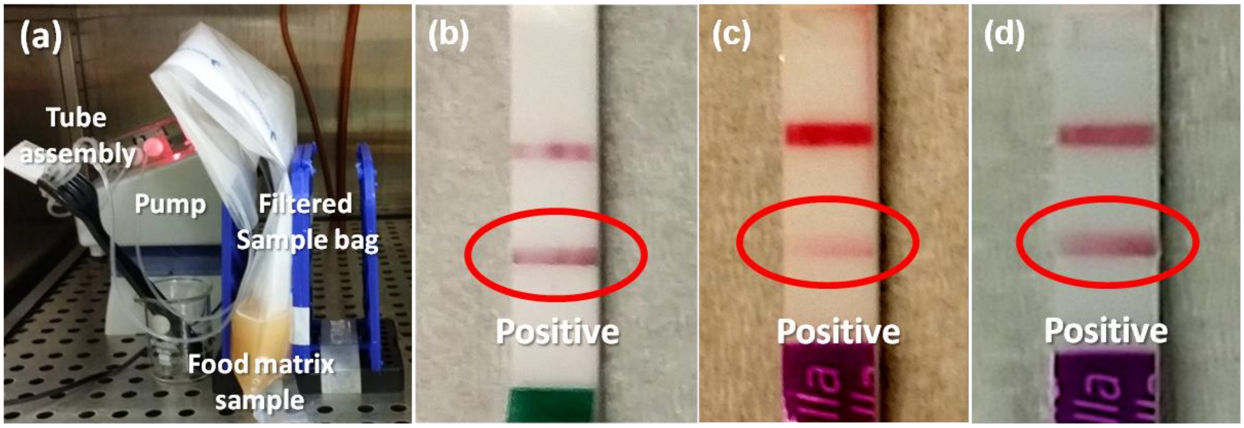

Figure S3. Positive detection by commercially available lateral flow immunoassay devices of S. typhimurium starting from 25 CFU in 250 mL containing 25 g ground chicken. (a) Positive results using Neogen Reveal 2.0 *Salmonella* strip in 14 hours in non-selective media.(b) Positive result using Romer Labs RapidChek SELECT *Salmonella* strip in 14 hours in non-selective media, (c) Positive result using Romer Labs RapidChek SELECT *Salmonella* strip in 9 hours in selective media

Application	Target recovery (Capture %)	Tube length	Flow rate	Time for a circulation of entire volume	# cycles of circulation	Total circulation time	#Ab/area
S. aureus 5 mL blood	80.3 ± 5.6 %	120 cm	0.5 mL/min	10 min	30	5 hours	~ $3x10^9$ /cm^2
MRSA 5 mL blood	95.4 ± 1.0 %	120 cm	0.5 mL/min	10 min	30		
MRSA 20 mL blood	96.5 ± 1.6 %	120 cm *	5 mL/min (1.25 mL/min in individual tube)	4 min	75		
S. aureus Rats *in vivo* (25 mL blood)	more than 2 Log_{10} reduction	240 cm	0.5 mL/min	50 min	N/A		
S. typhimurium Pure culture (100 mL)	56 - 92 %	120 cm *	6 mL/min (1.5 mL/min in individual tube)	16.7 min	22 - 25	6-7 hours	
S. typhimurium Pure culture (100 mL)	39.4 - 42.2 %	40 cm *		16.7 min	22 - 25		
S. typhimurium Food matrix (250 mL)	Qualitative only	120 cm *		41.7 min	9 - 10		

* The tube was split in four parts in order to reduce the flow rate.

Table S5. Summary of experimental parameters.

S4.1. The retention time within tube cannot be calculated in a straightforward manner, because (a) the proposed capturing system is based on constant flow of samples; (b) the sample volume differs by application (e.g. 5 mL - 20 mL for blood experiments and 100-250 mL for food experiments) while the tube's capacity is same (about 1 mL for both blood and food); (c) the quantity of bacteria is continuously increasing due to their growth during the process; (d) the bacteria's growth pattern differs due differences in sample (blood vs. food), bacteria strains, and initial concentrations. Considering the above, the number of cycles, i.e. the number of times the entire sample volume is circulated through the tube during a given time, is a more appropriate figure of merit.
